# The UK early access to medicines scheme: uptake, approvals, and reimbursement

**DOI:** 10.1017/S0266462325100421

**Published:** 2025-08-11

**Authors:** Christopher Felix Brewer

**Affiliations:** Cambridge, UK

**Keywords:** approval, drug, timelines, license, reimbursement mechanisms

## Abstract

**Objectives:**

The UK Early Access to Medicines Scheme (EAMS), launched in 2014, enables pre-license access to medicines for areas of high unmet medical need. This study aimed to evaluate the outcomes of the scheme by analyzing subsequent marketing authorization (MA), health technology assessment (HTA), and commissioning decisions.

**Methods:**

We conducted a retrospective analysis of all completed EAMS programs from 2014 to April 2025, reviewing MA, HTA, and commissioning outcomes.

**Results:**

Fifty-one EAMS programs were completed, over half in oncology. Median times from Scientific Opinion (SO) to MA, and reimbursement outcomes in England and Scotland were 4.3 (Q1: 2.6 and Q3: 7.3), 14.5 (Q1: 9.4 and Q3: 20.9), and 15.0 months (Q1: 11.4 and Q3: 18.1), respectively. Of 48 products appraised by the National Institute for Health and Care Excellence (NICE) or National Health Service (NHS) England, 50 percent received positive recommendations, 44 percent were optimized, and 6 percent were rejected. Of 45 products appraised by the Scottish Medicines Consortium or NHS Scotland, 73 percent were positive, 18 percent optimized, and 9 percent rejected. EAMS was qualitatively referenced in 48 percent of NICE appraisals and quantitatively in 18 percent.

**Conclusions:**

Compared to non-EAMS products, those entering the scheme achieve faster MA and HTA timelines and higher regulatory success. However, EAMS is referenced quantitatively in less than a fifth of NICE appraisals, and fewer than half of Promising Innovative Medicine designations progress to a full SO. Administrative burdens, data demands, and liability concerns may limit uptake; addressing these barriers could enhance the scheme’s impact.

## Key Points

- EAMS may facilitate faster access and higher reimbursement success rates
  Products that enter the UK Early Access to Medicines Scheme (EAMS) experience shorter timelines from Scientific Opinion (SO) to marketing authorization (MA) and health technology assessment (HTA), with higher rates of regulatory and reimbursement approval compared to non-EAMS products.
- Limited uptake and data integration
  Despite its benefits, EAMS is referenced in fewer than half of subsequent NICE appraisals, and only 18 percent include quantitative data from the scheme.
- Key barriers to broader impact
  Less than half of Promising Innovative Medicine designations progress to a full SO, potentially due to administrative burdens, evidentiary requirements, and liability concerns. Tackling these challenges may increase adoption and enhance the scheme’s overall value.

## Introduction

**Figure 1.**
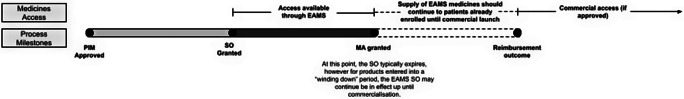
Key milestones in the EAMS process and relationship to medicines access. EAMS, Early Access to Medicines Scheme; MA, marketing authorization; PIM, Promising Innovative Medicine; SO, scientific opinion.

The UK Early Access to Medicines Scheme (EAMS) was introduced in 2014 by the Medicines and Healthcare products Regulatory Agency (MHRA) (1). The scheme aims to give patients with life-threatening or seriously debilitating conditions access to medicines that have not yet achieved marketing authorization (MA) where a clear unmet need can be demonstrated. To initiate an EAMS, pharmaceutical companies must first apply for a Promising Innovative Medicine (PIM) designation – awarded by the MHRA to products with early indications of suitability for the scheme based on available clinical and nonclinical data – followed by an application for a formal Scientific Opinion (SO) (1). The MHRA assesses each application to determine whether the new medicines are likely to offer significant advantages over existing therapies, while also outweighing potential risks. Once approved, the EAMS period can start (Figure 1).

Access to medicines during the EAMS period then typically follows a defined process. Approved SOs are published on the MHRA website, along with a public assessment report, protocol, and information for patients and the NHS. Healthcare practitioners are then able to contact the relevant manufacturing company to request access for patients who meet the criteria outlined in the protocol. EAMS medicines must be supplied free of charge to the NHS. The SO is valid for 1 year and will expire either at the end of this period (unless renewed) or upon the grant of an MA or extension of indication. However, a “winding down” period may be permitted following MA, allowing continued free-of-charge supply under the SO until the product is commercially available. After this point, routine patient access in the National Health Service (NHS) depends on the outcome of a reimbursement assessment – either a Health Technology Assessment (HTA) conducted by the National Institute for Health and Care Excellence (NICE) in England or the Scottish Medicines Consortium (SMC) in Scotland, or through NHS England and NHS Scotland commissioning policies when no formal HTA is undertaken.

If the respective HTA or commissioning body issues a positive recommendation, NHS organizations within that nation are then expected to fund and provide the medicine within the recommendation criteria. In contrast, a negative recommendation generally results in no provision of NHS-funded access unless an interim funding mechanism or individual funding request is approved. In such cases, it is at the manufacturer’s discretion whether to continue providing the medicine free of charge to patients who had previously accessed it through the EAMS program. For this reason, clear exit strategies addressing this scenario should be discussed with providers during the EAMS application process.

One of the core principles of EAMS is to provide access to new medicines in areas of unmet need while the regulatory and reimbursement review is undertaken. The latter process usually falls within the remit of NICE in England, who are a key stakeholder in the EAMS process (2). Once a PIM has been designated by the MHRA, NICE prioritizes the product for the topic selection process. In addition, the pharmaceutical company has multiple opportunities to meet with NICE during the EAMS process to ensure they are prepared for the Technology Appraisal (TA) or Highly Specialized Technologies Evaluation (HST) (1). These interactions allow companies to address uncertainties on key topics, such as anticipated positioning, clinical effectiveness, resource use, and safety, potentially using the EAMS process to collect data on any areas needing further clarification. Indeed, for pharmaceutical companies, it remains a priority to limit the risk of a negative reimbursement decision, not only due to the implications for patient inequity but also because of the potential ongoing obligation to supply treatment to patients already enrolled under the scheme.

Since its inception, there have been over 50 approved applications to the EAMS process (3). However, it does not represent the only route for healthcare practitioners to obtain access to new therapeutics in the absence of an MA (collectively deemed “early access”). In the European Union (EU) and the United Kingdom, unlicensed medicines can also be requested from the manufacturer and prescribed on a named patient basis (4;5). The main difference between EAMS and named patient use programs is that EAMS is a formal, structured process focused on regulatory and HTA engagement (which may include data collection), while named patient use programs provide individual access to unlicensed medicines on a case-by-case basis through a decentralized process, with limited formal oversight and public documentation. However, information from both programs can contribute to regulatory and HTA submissions.

This review aims to identify the products that have successfully completed an EAMS and to examine their subsequent regulatory and reimbursement outcomes. Evaluating this transition from early access to reimbursement is essential to understanding whether EAMS continues to function as a mutually beneficial mechanism – supporting timely access for patients, cost-effective care for the NHS, and a predictable pathway to market for pharmaceutical companies.

## Methods

To evaluate the timeliness and progression of EAMS products through their corresponding regulatory approval process, we examined the interval between SO approval and granting of MA. The details of all published expired EAMS SOs since inception in 2014 to 18 April 2025 were obtained from the MHRA website (6). For each product, data were extracted on the product name, EAMS indication, and dates of SO initiation and expiry. Information on MA, including dates of approval or variation, was sourced from the European Medical Agency (EMA) website for products licensed before 1 January 2021, during which the United Kingdom was still operating under EU regulatory frameworks (7). For products licensed after this date, data were obtained from the MHRA products database, regardless of whether approval was granted via the European Commission Decision Reliance Procedure or through the national licensing route (8). Additionally, aggregate data on PIM submissions and SO outcomes were collected from the MHRA website, with the latest available data cutoff used (November 2023) (3).

To assess how EAMS products perform in cost-effectiveness evaluations, we reviewed HTA outcomes, reimbursement decisions, and the time intervals with respect to SO and MA. The NICE and SMC websites were examined for information regarding HTA outcomes for the products submitted to the EAMS process in England and Scotland, respectively (2;9). For products that were not appraised by NICE or the SMC, commissioning outcomes were sourced from the NHS England and NHS Scotland websites (10;11). For each product, details were recorded on the assessment outcome, date of decision, and reimbursement indication.

In addition, NICE committee papers and final appraisal documents were individually searched for the keyword “EAMS” or “Early Access to Medicine Scheme” to identify qualitative or quantitative references.

All data extraction was conducted by a single author. All extracted data were organized and managed using Microsoft Excel spreadsheets. The data compiled, including details on EAMS SO, MA, and reimbursement outcomes, were obtained entirely from publicly accessible sources. Summary statistics were generated using Jamovi (Version 2.6) (12). For normally distributed data, results were presented as mean and standard deviation, while non-normally distributed data were reported as medians with corresponding lower (Q1) and upper (Q3) quartiles.

## Results

Between April 2014 and November 2023, the MHRA recorded a total of 171 PIM applications. Of these, 128 were granted, 24 refused, 4 withdrawn, and 15 remained pending at the time of data cutoff. During the same period, 65 SO applications were submitted, with 49 awarded, 4 refused, and 12 withdrawn.

Of the SOs granted through the EAMS pathway, 51 had expired by May 2024. The higher number recorded compared to the aggregated dataset above reflects the extended data cutoff used specifically for tracking EAMS expiries. These 51 expired SOs covered a broad range of therapeutic areas, with the highest representation in oncology (*n* = 28, 55 percent), followed by hematology (*n* = 5, 10 percent), dermatology (*n* = 4, 8 percent), genetic disease (*n* = 4, 8 percent), neurology (*n* = 4, 8 percent), infectious disease (*n* = 2, 4 percent), cardiology (*n* = 2, 4 percent), ophthalmology (*n* = 1, 2 percent), and autoimmune disease (*n* = 1, 2 percent). The median duration of operation of SO was 4.7 months (Q1: 2.3 and Q3: 9.0), with a relatively consistent number of applications submitted annually since the scheme’s inception in 2014 (Table 1).

**Table 1.** Characteristics and timelines of expired EAMS programs 2014–2024

Indication	*n* (%)
Oncology	28 (55)
Hematology	5 (10)
Dermatology	4 (8)
Genetic disease	4 (8)
Neurology	4 (8)
Infectious disease	2 (4)
Cardiology	2 (4)
Ophthalmology	1 (2)
Autoimmune disease	1 (2)

Duration (median)	Months (Q1, Q3)
SO awarded – SO expiry	4.7 (2.6, 9.0)
SO awarded – MA	4.3 (2.6, 7.3)
SO awarded – Reimbursement outcome (England)	14.5 (9.4, 20.9)
SO awarded – Reimbursement outcome (Scotland)	15.0 (11.4, 18.1)
MA – Reimbursement outcome (England)	10.1 (4.7, 15.6)
MA – Reimbursement outcome (Scotland)	8.4 (6.3, 12.3)
MA – NICE outcome	8.5 (4.7, 14.7)
MA – SMC outcome	8.6 (6.5, 12.4)

Year SO awarded	*n* (%)
2015	5 (10)
2016	6 (12)
2017	8 (16)
2018	3 (6)
2019	7 (14)
2020	8 (16)
2021	7 (14)
2022	5 (10)
2023	2 (4)

Abbreviations: IQR, interquartile range; MA, marketing authorization; NICE, National Institute of Clinical Excellence; SMC, Scottish Medicine Consortium; SO, scientific opinion.

Of the 51 products with expired SO, 47 (92 percent) went on to receive MA, either covering the full (*n* = 42) or part (*n* = 5) of the EAMS indication, while four (8 percent) had their MA applications withdrawn. The license restrictions included requirements for prior therapy exposure, higher-risk, or more advanced-stage disease, or a narrower subset of the population compared to the EAMS indication. Most products proceeded to reimbursement assessment in England (*n* = 48, via NICE or NHS England commissioning) and in Scotland (*n* = 45, via SMC or NHS Scotland commissioning), with 42 assessed by both HTA agencies.

The median time from granting of SO to published MA (based on the appropriate EMA or MHRA approval) was 4.3 months (Q1: 2.6 and Q3: 7.3). The median time from SO to reimbursement outcome was 14.5 months (Q1: 9.4 and Q3: 20.9) in England and 15.0 months (Q1: 11.4 and Q3: 18.1) in Scotland, reflecting NICE or NHS England commissioning and SMC or NHS Scotland commissioning outcomes, respectively. The median time between published MA and reimbursement outcome was 10.1 months (Q1: 4.7 and Q3: 15.6) in England and 8.4 months (Q1: 6.3 and Q3: 12.3) in Scotland.

**Figure 2.**
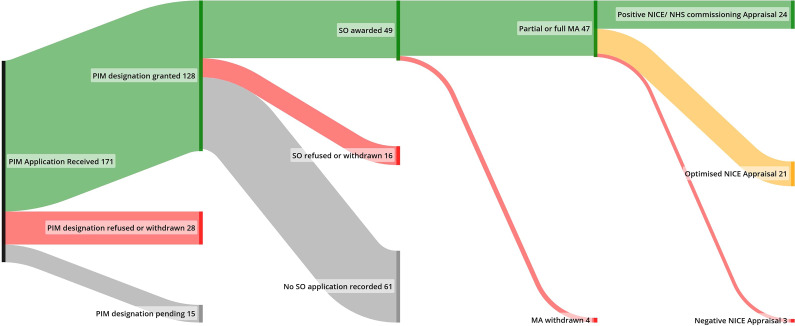
Flow of EAMS applications through to marketing authorization and reimbursement outcomes (NICE/NHS England). MA, marketing authorization; NICE, National Institute of Clinical Excellence; PIM, Promising Innovative Medicine; SO, scientific opinion.

The majority (*n* = 45) of EAMS products underwent a single TA or HST NICE appraisal, while three were evaluated within NHS commissioning policies, and three were not submitted or withdrawn before conclusion. Of those who underwent evaluation (*n* = 48), there were 24 (50 percent) positive, 21 (44 percent) optimized (with partial coverage of the EAMS indication or access through a managed access scheme or the cancer drugs fund), and 3 (6 percent) negative outcomes (Figure 2). Further details of optimized outcomes are shown in Table 2.

**Table 2.** Reimbursement outcomes and EAMS data utilization overview

NICE/NHSE commissioning outcome	*n* (%)
Positive	24 (50)
Optimized	
Managed access or CDF	8 (17)
Prior therapy restrictions	3 (6)
Stopping rules	7 (15)
Patient subgroup restrictions	3 (6)
Negative	3 (6)

SMC/NHS Scotland commissioning outcome	*n* (%)
Positive	33 (73)
Optimized	
Prior therapy restrictions	1 (2)
Stopping rules	2 (5)
Patient subgroup restrictions	5 (11)
Negative	4 (9)

Integration of EAMS data into NICE appraisals	
Level of integration	*n* (%)
No mention of EAMS	16 (36)
Referenced, but did not form part of the appraisal	11 (24)
Qualitative use	
Third-party experience	3 (7)
Demonstration of patient demand	7 (16)
Quantitative use	8 (18)

Abbreviations: CDF, cancer drugs fund; EAMS, Early Access to Medicine Scheme; NHSE, NHS England; NICE, National Institute of Clinical Excellence; SMC, Scottish Medicine Consortium.

**Figure 3.**
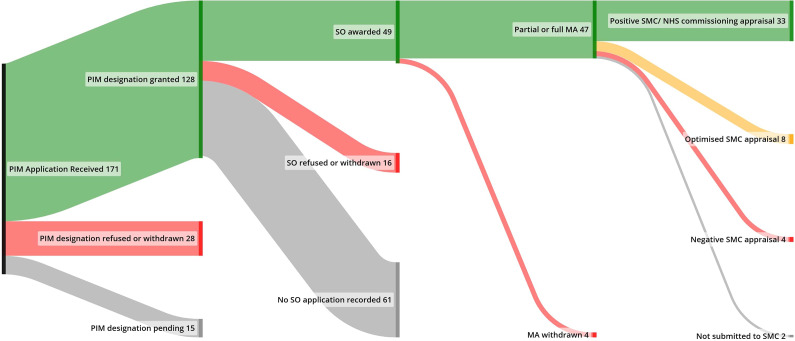
Flow of EAMS applications through to marketing authorization and reimbursement outcomes (SMC/NHS Scotland). MA, marketing authorization; PIM, Promising Innovative Medicine; SMC, Scottish Medicine Consortium; SO, scientific opinion.

A total of 44 of the 51 EAMS products with expired SO were submitted to the SMC, with one additional product undergoing review through NHS Scotland commissioning. Among the 45 products that underwent appraisal, 33 (73 percent) received a positive recommendation, 8 (18 percent) were accepted with an optimized recommendation, and 4 (9 percent) received a negative outcome (Figure 3).

There were 42 products that underwent HTA by both the SMC and NICE. The interval from MA to appraisal outcomes for these was similar: 8.5 months (Q1: 4.7 and Q3: 14.7) for NICE and 8.6 months (Q1: 6.5 and Q3: 12.4) for SMC. There were more negative recommendations for these products by the SMC than by NICE (4 vs. 2, respectively), although NICE was more likely to issue an optimized recommendation (20 vs. 8, respectively). Optimized recommendations were common across seven products and were usually associated with restrictions on the stage of disease or combination with other therapies.

Review of NICE committee papers and final appraisal documents found that EAMS was referenced in a total of 29 (64 percent) appraisals, with quantitative data used in just 8 (18 percent) (Table 2). Quantitative data included information extracted from EAMS regarding administration, demand, baseline patient characteristics, response rate, healthcare resource utilization, and safety.

## Discussion

Since its launch in 2014, EAMS has facilitated prelicense access to 51 medicines across a broad range of therapeutic areas, with oncology accounting for nearly half of all completed programs. Submissions to the scheme have remained steady over time, and the median duration of access under the approved SO was just under 5 months. Interestingly, fewer than half of all medicines that received a PIM designation went on to receive a positive SO. Potential causes for this discrepancy could include regulatory or commercial strategy shifts, clinical development setbacks, or challenges in preparing an SO application – such as limited internal capacity to meet the evidentiary or submission requirements.

Given that EAMS is designed to support access to treatments in areas of high unmet need – while enabling early engagement with both the MHRA and NICE – it is reasonable to expect that companies would prioritize candidates with strong regulatory and reimbursement potential. This strategy also helps mitigate the risk of patient inequity and reduces long-term liability for pharmaceutical sponsors, while also potentially expediting the approval process. The data support this approach: 92 percent of EAMS products subsequently secured and maintained MA during this period, compared to an estimated 85 percent of products that progressed through EMA appraisal between 2015 and 2025 (13).

Moreover, EAMS products received reimbursement outcomes faster following MA than those that had not engaged with NICE’s scientific advice. The average time from MA to reimbursement outcome in England was 10.1 months for EAMS products – comparable to previous research showing a 10.2-month interval for products that received prior NICE scientific advice, and notably shorter than the 13.3-month average for those that did not (14). Appraisal outcomes were also favorable: just 6 and 9 percent of EAMS products received a negative recommendation in England and Scotland, respectively. This compares well to broader figures from the literature, where ~20 percent of all cancer drug appraisals and 16 percent of all noncancer appraisals by NICE since 2020 resulted in non-recommendations (15;16). These findings may indicate that participation in EAMS encourages earlier or more proactive engagement with HTA bodies, which could, in turn, support faster and more favorable reimbursement outcomes.

Nonetheless, 44 percent of English and 18 percent of Scottish reimbursement outcomes for EAMS products were classified as “optimized,” meaning access was restricted to a narrower patient population than originally approved under the EAMS, or restricted for use within the cancer drug fund or through a managed access program. This demonstrates that, in certain circumstances, there may be a divergence between the patient populations defined in terms of clinical need for EAMS and those later deemed cost-effective by HTA bodies – a reflection of differing assessment strategies and priorities and the often limited data available at the time of the SO assessment. It also reinforces the importance of carefully defining the EAMS population – not only to reflect genuine unmet need but also to align with criteria likely to support a positive reimbursement decision.

The similar timing of appraisal outcomes by NICE and the SMC suggests a broadly aligned pace in assessing products approved under EAMS across the United Kingdom. However, the observed differences in recommendation types – more negative decisions by the SMC and more optimized recommendations by NICE – likely reflect underlying differences in appraisal criteria and methods, budget impact considerations, or healthcare priorities between Scotland and England.

Another important aspect of the EAMS program is its potential to facilitate the collection of data that can support subsequent reimbursement appraisals. While additional robust clinical data may be available at the time of HTA, EAMS offers scope for collecting UK-specific real-world evidence that may be valuable in the assessment, such as baseline patient characteristics within the medicine’s intended treatment population, response rates, tolerability, healthcare resource utilization, and safety, among other factors. However, this analysis found that fewer than a fifth of NICE appraisals for EAMS products included quantitative data derived from the program, while 48 percent featured only qualitative references. This may suggest that, in practice, EAMS does not consistently offer the scope or scale required to generate decision-informing evidence for HTA appraisal. Challenges, such as limited patient enrolment, short treatment durations, or delays in program initiation, may restrict the ability to collect robust real-world data in time for appraisal submissions.

Moreover, the modest data contribution from EAMS may reflect not only implementation-related limitations but also its relatively limited use as an early access route overall. A prior review of all publicly available NICE appraisal documentation for HTAs published between 1 January 2010 and 1 January 2021 (excluding those that were terminated, withdrawn, or replaced) found that over half (54.2 percent, i.e., 206 of 380 appraisals) referred to some form of expanded access, with 21.1 percent incorporating quantitative data (17). The fact that EAMS represents only a fraction of these cases underscores the potential broader underutilization of the program as a data-generating platform for reimbursement purposes. In addition, the EAMS process may not align with the long-term strategic planning of some pharmaceutical companies, particularly where global regulatory and market access strategies are prioritized over country-specific early access schemes.

Overall, these findings suggest that EAMS has, in many cases, fulfilled its intended purpose. The program has facilitated earlier access to promising treatments in areas of unmet clinical need, supported a relatively high rate of positive and timely reimbursement outcomes, and provided a framework that aligns with broader regulatory and HTA expectations. However, its limited uptake, variability in data contribution, and occasional misalignment between early access populations and those later deemed cost-effective highlight potential areas for improvement. Strengthening these aspects could help ensure that EAMS consistently delivers on all its objectives and better supports downstream reimbursement decisions.

Several limitations should be acknowledged in this analysis. First, all data were sourced from publicly available websites, which may not always be current or complete. As such, findings may be affected by potential inaccuracies or omissions in the underlying sources. Second, this analysis focused solely on medicines that progressed through the formal EAMS, excluding alternative unlicensed access routes in the UK – such as those granted on a named patient basis. Finally, it was not possible to fully assess the influence of quantitative or qualitative data from EAMS on the outcomes of NICE appraisals.

## Conclusions

Since its inception in 2014, a total of 51 EAMS programs have been completed, with submission rates remaining relatively consistent over time. Compared to non-EAMS products, those entering the scheme appear to benefit from faster MA and HTA timelines, along with higher rates of regulatory and reimbursement success.

Despite these advantages, EAMS still accounts for a relatively small proportion of early access activity overall and infrequently contributes quantitative evidence to NICE appraisals. This may reflect the complexity of the process, strategic priorities of pharmaceutical companies, concerns around sponsor liability, and the challenges of generating robust real-world data within the EAMS timeframe. Addressing these barriers may be essential to improving both the uptake and the broader utility of the scheme in supporting timely, evidence-based access to innovative treatments.
